# Anastrozole and everolimus in advanced gynecologic and breast malignancies: activity and molecular alterations in the PI3K/AKT/mTOR pathway

**DOI:** 10.18632/oncotarget.1799

**Published:** 2014-03-14

**Authors:** Jennifer J. Wheler, Stacy L. Moulder, Aung Naing, Filip Janku, Sarina A. Piha-Paul, Gerald S. Falchook, Ralph Zinner, Apostolia M. Tsimberidou, Siqing Fu, David S. Hong, Johnique T. Atkins, Roman Yelensky, Philip J. Stephens, Razelle Kurzrock

**Affiliations:** ^1^ Department of Investigational Cancer Therapeutics (Phase I Program), The University of Texas MD Anderson Cancer Center, Houston, TX; ^2^ Department of Breast Medical Oncology, The University of Texas MD Anderson Cancer Center, Houston, TX; ^3^ Foundation Medicine, Cambridge, MA; ^4^ Center for Personalized Cancer Therapy, Moores Cancer Center, University of California, San Diego, La Jolla, CA

**Keywords:** Anastrozole, Breast Cancer, Everolimus, Gynecologic Cancer, Hormone therapy

## Abstract

Background: Since PI3K/AKT/mTOR pathway activation diminishes the effects of hormone therapy, combining aromatase inhibitors (anatrozole) with mTOR inhibitors (everolimus) was investigated.

Patients and Methods: We evaluated anastrozole and everolimus in 55 patients with metastatic estrogen (ER) and/or progesterone receptor (PR)-positive breast and gynecologic tumors. Endpoints were safety, antitumor activity and molecular correlates.

Results: Full doses of anastrozole (1 mg PO daily) and everolimus (10 mg PO daily) were well tolerated. Twelve of 50 evaluable patients (24%) (median = 3 prior therapies) achieved stable disease (SD) ≥ 6 months/partial response (PR)/complete response (CR) (n = 5 (10%) with PR/CR): 9 of 32 (28%) with breast cancer (n=5 (16%) with PR/CR); 2 of 10 (20%), ovarian cancer; and 1 of 6 (17%), endometrial cancer. Six of 22 patients (27%) with molecular alterations in the PI3K/AKT/mTOR pathway achieved SD ≥ 6 months/PR/CR. Six of 8 patients (75%) with SD ≥ 6 months/PR/CR with molecular testing demonstrated at least one alteration in the PI3K/AKT/mTOR pathway: mutations in PIK3CA (n=3) and AKT1 (n=1) or PTEN loss (n=3). All three responders (CR (n = 1); PR (n=2)) who had next generation sequencing demonstrated additional alterations: amplifications in CCNE1, IRS2, MCL1, CCND1, FGFR1 and MYC and a rearrangement in PRKDC.

Conclusions: Combination anastrozole and everolimus is well tolerated at full approved doses, and is active in heavily-pretreated patients with ER and/or PR-positive breast, ovarian and endometrial cancers. Responses were observed in patients with multiple molecular aberrations.

Clinical Trails Included: NCT01197170

## INTRODUCTION

Estrogens regulate growth, differentiation and development in many tissues including the female reproductive tract, bone, central nervous system, immune and cardiovascular systems [1-3]. The actions of estrogens are mediated by a group of estrogen receptors (ER). Aromatase, a member of the cytochrome P450 superfamily, catalyzes the final step in the biosynthesis of estrogen from androgen [4]. In women with breast cancer, the expression of aromatase is the highest in or near tumor sites [5, 6]. Anastrozole (Arimidex) is a potent nonsteroidal aromatase inhibitor, producing approximately 97% inhibition of estrogen biosynthesis [7].

Several studies have evaluated combination approaches using hormone therapy with other targeted agents in patients with breast and gynecologic cancers. The Bolero-2 trial (trial registration ID: NCT00863655), a large randomized Phase III study, compared the aromatase inhibitor exemestane alone to a combination of exemestane with everolimus, an mTOR inhibitor [8]. The combination improved progression-free survival (PFS) in patients with ER and/or progesterone receptor (PR)-positive advanced and metastatic breast cancer. Based on data from the Bolero-2 trial, in July 2012 the US Food and Drug Administration (FDA) approved everolimus in combination with exemestane for use in postmenopausal women with hormone receptor-positive, HER2/*neu*-negative, advanced breast cancer who have progressed on anastrozole or letrozole. Other studies have evaluated the aromatase inhibitor letrozole in combination with everolimus and have demonstrated responses in patients with metastatic endometrial carcinoma (trial registration ID: NCT01068249) [9] and breast cancer (trial registration ID: NCT00107016) [10].

Research into the mechanism of endocrine responsiveness and resistance in breast and gynecologic cancers has revealed that the PI3K/AKT/mTOR pathway becomes activated and utilized by cancer cells to bypass the effects of hormone therapy [11-13]. We therefore investigated the use of the aromatase inhibitor anastrozole in combination with everolimus in patients with ER and/or PR-positive breast and gynecologic tumors including ovarian and endometrial cancer (trial registration ID: NCT01197170) and including an analysis of molecular data.

## RESULTS

### Patients

Fifty-five women with advanced or metastatic breast, ovarian, endometrial, and cervical malignancies were enrolled. All patients received at least one dose of treatment. All patients were considered eligible for toxicity evaluation. Fifty patients were considered eligible for response evaluation. Five patients were ineligible for response evaluation because they had not reached restaging at the time of analysis. Demographic and clinical characteristics of evaluable patients are summarized in Table 1. The median age of patients was 57 years (range: 24-82 years). The median number of prior therapies in the advanced or metastatic setting was 3 (range: 0-13). Twenty-three of the evaluable patients had received prior aromatase inhibitors. Fifteen deaths occurred, all attributed to disease progression. Expansion cohorts were opened for breast, endometrial, ovarian and cervical tumor types and for patients with PTEN loss and *PIK3CA* mutations.

**Table 1:** Patient characteristics

Characteristics	No. of Evaluable Patients	%
**Race/ethnicity**	**50**	
White	45	90
Black	1	2
Hispanic	3	6
SW Asian	1	2
**Age (years)**		
Median (range)	57 (24-82)	
**Previously treated with aromatase inhibitors**		
Median (range)	1 (0-3)	
**No. of prior therapies in metastatic setting**		
Median (range)	3 (0-13)	
**ECOG performance score**		
0	11	22
1	32	64
2	7	14
**Primary organ site**		
**Uterine**	**6**	**12**
Endometrial	5	10
Mullerian	1	2
**Ovarian**	**10**	**20**
Epithelial	9	18
Stromal	1	2
**Cervix**	**2**	**4**
**Breast**	**32**	**64**

### Overall Survival and Time to Treatment Failure

The median survival has not been reached after a median follow up of 6.1 months. At the time of analysis, 37 of 55 (67%) were off study. The overall median TTF was 3.1 months (95% CI 2.1-4.1).

### Dose Escalation, DLT and Tolerance

Seven patients were enrolled at dose level 1 and 48 at dose level 2. Two of 55 patients (4%) experienced a DLT. The two DLTs both occurred in expansion cohorts of dose level 2 and were grade 3 mucositis. The full federal drug administration (FDA) dose for each drug evaluated in dose level 2 (anastrozole 1 mg PO daily and everolimus 10 mg PO daily) was found to be safe and well tolerated.

Twenty-five of 55 patients (45%) experienced at least one drug-related toxicity. Of the 36 reported drug-related toxicities, 25 (69%) were grade 1 or 2. The most common grade 1 and 2 drug-related toxicities included mucositis (6 patients), fatigue (4 patients), nausea/vomiting/anorexia, elevated cholesterol, pneumonitis, elevated triglycerides and elevated ALT (2 patients each). There were 11 grade 3 toxicities at least possibly related to treatment including mucositis (2 patients), pneumonitis, hypertension, hyperglycemia, hemoptysis, weakness, rash, low platelets, elevated AST and decreased ANC (1 patient each). A dose modification was required in four incidents for mucositis (2 patients), nausea (1 patient), and pneumonitis (1 patient). Two patients with pneumonitis (including one with grade 2 and one with grade 3 toxicity) were taken off study with resolution of toxicity.

### Response Data

Twelve of 50 evaluable patients (24%) achieved SD ≥ 6 months/PR/CR, including 5 patients (10%) with PR/CR: 9 of 32 patients (28%) with breast cancer (cases 1, 2, 3, 4, 5, 6, 9, 10 and 12, Table 2); 2 of 10 patients (22%) with ovarian cancer (cases 7 and 11, Table 2); and 1 of 6 patients (17%) with endometrial cancer (case 8, Table 2). Neither of the 2 patients with cervical cancer achieved SD≥6 months/PR/CR. Five patients with breast cancer achieved a PR (cases 3, 4 and 5, Table 2) or CR (cases 1 and 2, Table 2). Three patients with PR included one patient with a 50% decrease in disease for 11 months (case 3, Table 2), one patient with a 44% decrease in disease for 2 months (case 4, Table 2) and, one with a 38% decrease in disease for 17+ months (case 5, Table 2). The two patients (4%) with CRs have ongoing responses at 9+ and 6+ months (cases 1 and 2, Table 2).

**Table 2:** Response characterization by patient

Case No.	Age/ Sex	Disease site	Histology	ER status	PR status	HER2/*neu* status^A^	No. Prior Treatments^B^	Prior Aromatase Inhibitors^B^	Best response (%)	TTF (months)	Molecular CLIA results other than NGS^C^	NGS testing^E^	NGS positive results
**1**	44/F	Breast	Ductal	Positive	Negative	Negative	2	Letrozole (4 months)	CR	9+	PTEN present (IHC)	Yes	TP53 mutation CCNE1 amplification IRS2 amplification MCL1 amplification
**2**	70/F	Breast	Lobular	Positive	Positive	Negative	2	Anastrozole (12 months) Exemestane (13 months)	CR	6+	Not Done	No	Not done
**3**	38/F	Breast	Ductal	Positive	Positive	Negative	4	None	PR (-50%)	11	PTEN absent (IHC)	Yes	CCND1 amplification FGFR1 amplification PRKDC re-arrangement
**4**	82/F	Breast	Lobular	Positive	Negative	Negative	10	Letrozole (12 months) Anastrozole (1 month) Exemestane (2 months)	PR (-44%)	2	Not Done	No	Not done
**5**	48/F	Breast	Ductal	Positive	Positive	Negative	7	None	PR (-38%)	15+	PTEN present (IHC) PIK3CA+, BRAF-, EGFR-, NRAS-, KRAS-, GNAQ-, c-KIT-, MET-	Yes	PIK3CA mutation PIK3R1 mutation CCND1 amplification FGFR1 amplification MYC amplification
**6**	46/F	Breast	Ductal	Positive	Positive	Negative	7	None	SD (-21%)	7	PTEN present (IHC) KRAS+, MET- AKT1-, c-KIT- BRAF-, EGFR- NRAS-,PIK3CA-	No	Not done
**7**	49/F	Ovarian	Serous	Positive	Positive	Unknown	5	None	SD (-19%)	6	Not Done	No	Not done
**8**	64/F	Uterine	Endometrial	Positive	Positive	Unknown	3	None	SD (-19%)	7+	PTEN absent (IHC) KRAS+ AKT1-, BRAF- GNAQ-, GNAS- IDH1-, IDH2- MET-, NRAS- PIK3CA-, RET-	No	Not done
**9**	54/F	Breast	Ductal	Positive	Negative	Negative	9	Anastrozole (36 months) Exemestane (7 months)	SD (-16%)	7	Not Done	No	Not done
**10**	60/F	Breast	Ductal	Positive	Positive	Negative	1	Anastrozole (2 months)	SD (-6%)	10+	PTEN present (IHC) PIK3CA+	No	Not done
**11**	60/F	Ovarian	Serous	Positive	Negative	Unknown	2	None	SD (-5%)	15	PTEN absent (IHC) PIK3CA-, EGFR- KRAS-	No	Not done
**12**	73/F	Breast	Lobular	Positive	Negative	Positive	0	None	SD (0%)	7+	AKT1+, PIK3CA+^D^	No	Not done

A **Her2/neu positive patients were confirmed by IHC and/or FISH analysis**

B **In the metastatic setting**

C **All CLIA testing at MDACC were for mutations except where indicated by (IHC); (+) denotes mutation and (-) denotes wild-type**

D **Molecular testing performed at by sequencing (CLIA-certified) at Knight Diagnostics (Portland, OR, USA) (patient #12). Panel available at http://www.ohsu.edu/xd/health/services/cancer/getting-treatment/services/knight-diagnostic-laboratories/kdl-test-offering-list.cfm**

E **NGS performed at Foundation Medicine (Cambridge, MA, USA)**

### Prior Treatment with Aromatase Inhibitors and Response

Twenty-three of 50 evaluable patients (46%) had received at least one prior aromatase inhibitor in the advanced or metastatic setting. Five of the 23 patients (22%) who had been previously treated in the metastatic setting with an aromatase inhibitor achieved SD ≥ 6 months/PR/CR with the combination of anastrozole and everolimus, including 3 patients (13%) with PR/CR. Twenty of 32 patients (63%) with breast cancer had received prior aromatase inhibitors in the advanced or metastatic setting. Five of the 20 patients (25%) with breast cancer and prior aromatase inhibitor exposure achieved SD ≥ 6 months/PR/CR (3 patients with PR/CR).

### Molecular Analysis and Association with Response

When archival cell blocks for patients were available, CLIA-certified molecular testing was performed for PI3K/AKT/mTOR pathway alterations. For the purposes of this study, we defined a PI3K/AKT/mTOR pathway alteration as one or more of the following: *PIK3CA*, *AKT1*, *PTEN* mutation, and/or PTEN loss (by IHC). Of the 12 patients who demonstrated SD ≥ 6 months/PR/CR, 8 had molecular testing (3 of 5 with PR/CR). Six of the 8 patients (75%) had at least one alteration in the PI3K/AKT/mTOR pathway including *PIK3CA* mutations (3 patients, one of whom also had an *AKT1* mutation) and PTEN loss (IHC) (3 patients). The remaining two patients (25%) with molecular testing did not have a direct alteration in this pathway. Three patients who achieved PR/CR who also had molecular testing with NGS demonstrated additional alterations: amplifications in *FGFR1* (encodes for fibroblast growth factor receptor 1, 2 patients), *CCND1* (encodes for cyclin D1, also known as *BCL1*, 2 patients), *CCNE1* (encodes for cyclin E1, 1 patient), *IRS2* (encodes for insulin receptor substrate 2, 1 patient), *MCL1* (myeloid leukemia cell gene, 1 patient) and *MYC* (myelocytomatosis viral oncogene, 1 patient) and, a re-arrangement in *PRKDC* (protein kinase DNA activated catalytic polypeptide, 1 patient).

A total of 35 patients had molecular testing for at least one of the following: *PIK3CA, PTEN*, or *AKT1* mutation; and/or PTEN loss. Of the 35 patients tested, 22 (63%) were positive for at least one alteration in the PI3K/AKT/mTOR pathway. Of 35 patients tested for an alteration in the PI3K/AKT/mTOR pathway, 13 were negative. One of 13 patients (8%) attained SD ≥ 6 months/PR/CR (this patient had a CR). By comparison, 22 patients tested had a PI3K pathway alteration; 6 of these 22 patients (27%) had SD≥6 months/PR/CR (2 patients with PR/CR) (*p* = 0.16).

## DISCUSSION

Hormonal therapy is a mainstay of treatment for breast cancer and is an area of active investigation in gynecologic tumors. Strategies to augment response and overcome resistance to aromatase inhibitors are urgently needed. PI3K/AKT/mTOR pathway alterations are common in breast and gynecologic cancers [20-22]. Preclinical studies have shown that suppression of PTEN function, or activated AKT1 expression, caused by activating mutations in PIK3CA or AKT1, confers resistance to traditional chemotherapeutic drugs as well as hormonal based drugs, but results in sensitivity to mTOR inhibitors [23, 24]. Recent studies of breast cancer patients treated with everolimus in combination with exemestane [8] and of endometrial and breast cancer patients treated with everolimus in combination with letrozole [9, 10] have shown efficacy. Based on the Phase III study results, everolimus is now FDA-approved in combination with the aromatase inhibitor exemestane in patients with advanced, hormone receptor-positive breast cancer refractory to anastrozole and/or letrozole [8].

Our study indicates that the combination of anastrozole and everolimus can be given at full approved doses. Tolerance was excellent with the main side effects being grade 1 and 2 mucositis, fatigue, nausea/vomiting/anorexia and, grade 3 mucositis. Two patients experienced DLTs, mucositis in both cases. A dose reduction resulted in better tolerance in one patient and the other patient was taken off study. These results parallel those for exemestane [8] and letrozole [9, 10]; these hormone antagonists can be given safely with full dose everolimus (10 mg PO daily).

Herein we report that 6 of 8 patients (75%) who experienced SD ≥ 6 months/PR/CR (3 patients with PR/CR) on this study and on whom molecular testing was performed, demonstrated at least one alteration in the PI3K/AKT/mTOR pathway. Six of 22 patients (27%) with molecular alterations in the PI3K/AKT/mTOR pathway achieved SD ≥ 6 months/PR/CR, including 2 patients (9%) with PR/CR. Our results support and expand on those previously reported [21, 25]. Indeed, 12 of 50 heavily pretreated patients (24%) with breast or gynecologic tumors achieved SD ≥ 6 months/PR/CR with anastrozole and everolimus, including 5 patients (10%) with PR/CR. These results are consistent with the SD ≥ 6 months/PR/CR rates of 20 to 30% previously reported by our group in patients treated with matched phase I therapy [21, 25]. Our response rates are slightly higher than those reported in Bolero-2 [8], but our patient numbers are also smaller. Further, some of our patients with breast cancer did not receive prior aromatase inhibitors. The number of patients with endometrial cancer are small, but the observed activity is lower than reported in the letrozole/everolimus combination in this tumor type [9]. Regarding correlation with molecular aberrations, we found that six of eight responders tested had pathway aberrations. These aberrations included abnormalities in *PIK3CA*, *PTEN* and *AKT*, consistent with the diversity of genes that can activate the PIK3CA/AKT/mTOR pathway. On the other hand, only 27% of patients with these pathway abnormalities responded. These data are consistent with data recently presented by Hortobagyi et al. [26], where patients with either no or only one molecular aberration in the PI3K/AKT/mTOR pathway had better outcomes with exemestane and everolimus than did patients with multiple aberrations. On the other hand, three of our patients with CR or PR showed multiple aberrations indicating that their presence does not rule out response.

A total of 3 patients who achieved PR/CR had NGS testing. These patients demonstrated numerous alterations including: *CCNE1*, *CCND1*, *FGFR1*, *IRS2*, *MCL1*, and *MYC* amplifications and a *PRKDC* re-arrangement, as well as *TP53* mutations (cases 1, 3 and 5, Table 2). *CCNE1* and *CCND1* code for cyclin D1 and cyclin E1, respectively, which are proteins that help control the transition of cells from G1 to S phase during proliferation [27]. Both of these proteins are regulated by GSK-3β, which is directly phosphorylated by AKT [28]. *MYC* is a regulator gene that codes for a transcription factor involved in cell proliferation and is also directly regulated by GSK-3β [29]. Insulin receptor substrates (IRS1 and IRS2) are proteins that dock to IGFR receptors to recruit other factors such as the p85 regulatory subunit of PI3K, thereby leading to activation of the PI3K/AKT/mTOR pathway [30]. Fibroblast growth factor receptors (FGFR) bind to growth factors (FGF) involved in angiogenesis. Activation of FGFR induces PI3K and AKT activities through recruitment and tyrosine phosphorylation of the docking protein Gab1 that results in the activation of PI3K [31]. *MCL1* codes for a protein that enhances cell survival by inhibiting apoptosis. Control of MCL1 stability by GSK-3β is an important mechanism for the regulation of apoptosis by AKT [32]. Tumor Protein 53 (*TP53*) codes for a tumor suppressor (p53) that regulates cell cycle. AKT influences the activity of p53 through phosphorylation of the p53-binding protein MDM2 [33]. Protein kinase, DNA-activated, catalytic polypeptide (PRKDC) codes for a catalytic subunit of the DNA-dependent protein kinase, a member of the PI3K family [34]. The presence of multiple gene aberrations may reflect increased genetic instability and poor patient prognosis [35]; however, the combination of anastrozole and everolimus was beneficial for each of these three patients. One of the patients with a durable CR (case 1, Table 2) had NGS testing and demonstrated a *TP53* mutation as well as amplifications in *CCNE1*, *IRS2*, a protein important for insulin receptor signaling [36], and *MCL1*, a gene that plays a role in regulating cell-fate decisions [37]. The latter results suggest that CR can be achieved in the absence of a direct alteration in the PI3K/AKT/mTOR pathway, even when multiple molecular aberrations are present. However, some of the above genes may modulate the PIK3CA/AKT/mTOR axis perhaps explaining in part the response [31, 38-43]. Systems biology bioinformatics approaches will be needed to determine whether or not multiple aberrant signals in patients with metastatic tumors converge on pathways such as PI3K/AKT/mTOR pathway, and are hence actionable.

Our results suggest that patients can obtain salutary effects including CR (and PR) despite the presence of multiple alterations; however, should relapse occur in any of these patients, it might be explained by the additional alterations eventually leading to resistance. It is not surprising that patients with advanced disease have multiple molecular alterations, as this observation is consistent with previous literature reports of heterogeneity within and between tumors [44-46].

**Table 3:** Response by disease type and histology

Disease site histology	Evaluable patients	CR (%)	PR (%)	SD≥6 months	SD≥6 months/PR/CR
**Uterus**	**6**				
Endometroid adenocarcinoma	5			1	1
Mullerian^A^	1				
**Ovary**	**10**				
Serous	6			2	2
Endometroid adenocarcinoma	3				
Granulosa Cell	1				
**Cervix**	**2**				
Adenocarcinoma	1				
Small Cell	1				
**Breast**	**32**				
Ductal	24	1	2	3	6
Lobular	5	1	1	1	3
Lobular/Ductal	1				
Papillary	1				
Papillary/Mucinous	1				

A Tumor of this patient had equivalent high grade carcinoma and sarcoma components and contained ovarian remnants.

There are several limitations to our study. Whether the responses observed are due simply to the inhibition of the PI3K/AKT/mTOR pathway by everolimus or due to hormone modulation by aromatase inhibition alone (especially in those patients not previously treated with anastrozole) or contribution from both is unknown, as this was not a randomized study. However, it should be noted that the rate of SD ≥ 6 months/PR/CR was similar in patients who had failed prior aromatase inhibition therapy (5 of 23 patients, 22%) versus those who had not had aromatase inhibition (7 of 27 patients, 26%), which suggests that everolimus contributed to those responses. Another potential limitation of this analysis is that a majority of our patients had breast cancer, and that the combination of an aromatase inhibitor and an mTOR inhibitor has recently been demonstrated to be effective. However, we provide correlative molecular data and show responses in endometrial and ovarian cancers, as well as breast cancer. Further, we show that the activity in breast cancer is seen with anastrozole and therefore is not limited to exemestane (as used in previous studies) [8]. Additional limitations of our study stem from the fact that only a subset of patients had next generation sequencing. Furthermore, comparison of MD Anderson CLIA “hotspot” testing versus next generation sequencing was also performed in only limited number of patients. It is possible that other mutations exist in some of the patients tested by “hotspot” analysis, and were not discerned by this type of testing. As an example, a recent study in breast cancers has identified additional driver mutations in AKT1 that are not located in hotspot regions frequently tested [47]. We also had molecular data from only one time point; pre- and post-tissue analyses would be more informative to elucidate resistance patterns. Future investigations should emphasize molecular analysis so that biological correlates of clinical response can be identified. Finally, our patient numbers were relatively small. Therefore, these results must be interpreted in this context and considered hypothesis generating.

In conclusion, our study demonstrates that full doses of the aromatase inhibitor anastrozole and the mTOR inhibitor everolimus can be given for prolonged periods of time (longest so far = 17 months) with excellent tolerance. A subset of heavily pretreated patients with breast, ovarian and endometrial cancer achieved SD ≥ 6 months/PR/CR. SD ≥ 6 months/PR/CR was seen in 27% of patients with molecular alterations in the PI3K/AKT/mTOR pathway. Patients with multiple molecular alterations still benefited from therapy. Further exploration of this combination in larger cohorts of patients with breast and gynecologic tumors and in-depth analysis of molecular correlates is warranted.

## PATIENTS AND METHODS

### Patients

The study enrolled adult patients with pathologically-confirmed advanced or metastatic cancer. Patients were required to have tumors that demonstrated ER and/or PR-positive disease (positivity by immunohistochemistry staining ≥ 1% based on reported guidelines for breast cancer) [14]. For women, only those who were postmenopausal or premenopausal and receiving a gonadotropin-releasing hormone agonist were eligible. All patients had an Eastern Cooperative Oncology Group (ECOG) performance status score of 0 to 2. Other criteria included adequate neutrophil counts (≥ 1,000/mL), platelets (≥ 50,000/mL), creatinine (≤ 2 × upper limit of normal), bilirubin (≤ 2.0), alanine aminotransferase (ALT) (≤ 3 × upper limit of normal with the exception of patients with liver metastasis in whom ALT ≤ 5 × upper limit of normal and bilirubin ≤ 3 × upper limit of normal). Patients provided written informed consent according to institutional guidelines. The study was approved by the Institutional Review Board (IRB).

### Study Design and Treatment

This was a single institution, open-label, dose-escalation study, with a standard 3+3 design. The study allowed an additional (optional) three patients at any dose level in order to provide flexibility for enrollment and additional safety or correlative data. Endpoints were to establish safety and preliminarily assess antitumor efficacy and molecular correlates.

Each treatment cycle was 28-days with both drugs given daily. Cycles were consecutive with no treatment breaks. Patients were initially treated at dose level 1 (anastrozole 1 mg PO daily and everolimus 5 mg PO daily) and if no serious toxicities were observed the dose was escalated to dose level 2 (anastrozole 1 mg PO daily and everolimus 10 mg PO daily). Dose level 2 represents the full approved dose of each drug. Once the safety of dose level 2 was established, additional patients were enrolled at that level.

If a response was observed in a particular tumor subtype, study cohorts were expanded to include up to an additional 14 participants with that specific tumor type. For the purpose of adding up to 14 additional participants, a tumor (including, but not limited to tumor type, histologic subtype or genomic subtype) response was defined as one of the following: (1) stable disease (SD) ≥ 6 months; or (2) decrease in measurable tumors ≥ 20% by RECIST criteria.

### Assessment of Safety and Efficacy

Treatment continued until unacceptable toxicity or disease progression occurred. Dose delays and reductions were at the discretion of the treating physician. Toxicities were graded based on the Common Terminology Criteria for Adverse Events (CTCAE v4.0). Dose limiting toxicities (DLTs) were defined as adverse events (AEs) related to study agents that occurred during the first cycle of treatment with an attribution of possible, probable, or definitely related to therapy and fulfilling one of the following requirements: grade 4 neutropenia lasting > 7 days, febrile neutropenia, platelet count <25,000/mm lasting >7 days, any grade 4 or toxicity, any grade 3 toxicity (excluding nausea, vomiting and diarrhea unless it persisted despite optimal treatment).

Responses were assessed after three cycles (about 12 weeks) or earlier at the discretion of the treating physician. All radiological tests were assessed by an MD Anderson radiologist. In addition, results were reviewed in a departmental tumor measurement clinic and by the attending physician. RECIST criteria were used for progressive disease (PD), stable disease (SD), partial and complete responses (PR and CR).

### Molecular Assays for Biological Markers: PIK3CA, AKT1, PTEN mutations, and PTEN loss

Clinical Laboratory Improvement Amendment (CLIA) certified mutational and/or immunohistochemistry assays were performed, when tissue was available, for *PIK3CA*, *AKT1*, and *PTEN* mutations, and PTEN expression. The tests were done within the Division of Pathology and Laboratory Medicine at MD Anderson. Archival formalin-fixed, paraffin-embedded tissue blocks or tissue from fine-needle aspiration or surgical biopsies were used for mutational analysis. DNA was extracted and analyzed using a polymerase chain reaction (PCR)-based DNA sequencing method for *PIK3CA* mutations in codons [c] 532-554 of exon 9 (helical domain) and c1011-1062 of exon 20 (kinase domain) [15], which included the mutation hot spot region of the *PIK3CA* proto-oncogene by Sanger sequencing following amplification of 276 bp and 198 bp amplicons, respectively, utilizing primers designed at MD Anderson. For *AKT1* and *PTEN* mutations, similar methods were used. Codons 17, 1173, and 179 were examined for *AKT1* mutations, and for *PTEN,* the entire coding sequence of exons 1 through 9 were examined as previously described [16]. PTEN loss was assessed using a Dako antibody (Carpinteria, CA, USA) as previously published [16, 17].

### Evaluation of HER2/neu amplification, estrogen and progesterone receptor status

Under CLIA conditions, immunohistochemistry was used to measure of HER2/*neu*, estrogen and progesterone receptors. Estrogen and progesterone receptors were assessed using antibody 6F11 (Novocastra Laboratories, Ltd., Newcastle Upon Tyne, UK). Alternatively, fluorescence in situ hybridization (FISH) was used to measure the copy number of HER2/*neu*.

### Next-Generation Sequencing

Genomic libraries were captured for 3230 exons in 182 cancer-related genes plus 37 introns from 14 genes often rearranged in cancer and sequenced to average median depth of 734× with 99% of bases covered >100× (Foundation Medicine, Cambridge, MA, USA).

### Statistical Analysis

**Fig 1:**
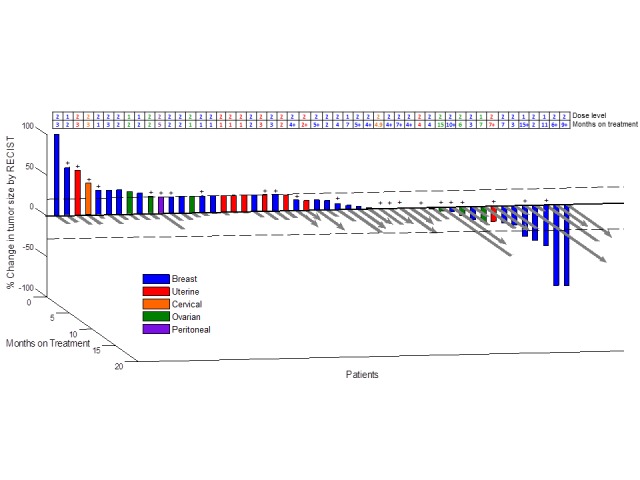
3D Waterfall Best radiologic response by RECIST and months on treatment (N = 50 patients). (+) Patients positive for alterations in PI3K/AKT1/mTOR pathway.

Descriptive statistics are provided for all endpoints using SPSS v.19 (Chicago, IL, USA). Continuous measurements were summarized using mean, standard deviation, median, range, number of patients, and percentages. Time to treatment failure (TTF) and overall survival were calculated using the method of Kaplan and Meier [18, 19] in months, from date of first treatment dose to the date of last treatment dose or death from any cause, whichever came first. Patients still on study at the time of data analysis were censored at the time of last assessment for TTF. For survival, patients still alive at the time of analysis were censored at that time point. A three-dimensional waterfall plot depicting best RECIST responses by percent and time on therapy is presented in Figure 1.
